# High-Efficiency Drug Loading in Lipid Vesicles by MEMS-Driven Gigahertz Acoustic Streaming

**DOI:** 10.3390/mi16050562

**Published:** 2025-05-07

**Authors:** Bingxuan Li, Haopu Wang, Zhen Wang, Huikai Xie, Yao Lu

**Affiliations:** 1 School of Integrated Circuits and Electronics, Beijing Institute of Technology, Beijing 100081, China; 3120221321@bit.edu.cn (B.L.); haopu.wang@bit.edu.cn (H.W.); wangzhenqh@163.com (Z.W.); 2 Engineering Research Center of Integrated Acoustic-Opto-Electronic Microsystems (Ministry of Education of China), Beijing 100081, China; 3 Chongqing Institute of Microelectronics and Microsystems, Beijing Institute of Technology, Chongqing 400030, China

**Keywords:** acoustic streaming, gigahertz, MEMS, drug encapsulation, vesicles

## Abstract

Drug carriers hold significant promise for precision medicine but face persistent challenges in balancing high encapsulation efficiency with structural preservation during active loading. In this study, we present a microelectromechanical system (MEMS)-driven platform that can generate gigahertz (GHz)-frequency acoustic streaming (1.55 GHz) to enable nondestructive, power-tunable drug encapsulation in lipid vesicles. Utilizing DSPE-PEG-modified bilayers with hydrodynamic shear forces, our method achieves transient membrane permeability that preserves membrane integrity while permitting controlled doxorubicin (DOX) influx. We developed the GHz acoustic MEMS platform and applied it to systematically investigate two drug loading strategies: (1) loading DOX into giant unilamellar vesicles (GUVs, >10 μm in diameter) prior to extrusion into small unilamellar vesicles (SUVs, 100 nm) versus (2) direct acoustic loading into pre-formed SUVs. The GUV-first approach demonstrated better performance, achieving 60.04% ± 1.55% encapsulation efficiency (EE%) at 250 mW acoustic power—a 5.93% enhancement over direct SUV loading (54.11% ± 0.72%). Structural analysis via TEM confirmed intact SUV morphology post-loading, while power-dependent EE% analysis showed a linear trend. This work bridges gaps in nanocarrier engineering by optimizing drug loading strategies, aiming to offer a potential drug carrier platform for drug delivery in biomedical treatment in future.

## 1. Introduction

Drug delivery systems have revolutionized therapeutic strategies by enabling precise control over pharmacokinetics and spatiotemporal drug release, bridging the gap between conventional pharmacology and precision medicine [1]. Central to these systems are engineered drug carriers capable of controlled drug release. Among engineered nanocarriers, small unilamellar vesicles (SUVs) have emerged as indispensable nanocarriers in precision drug delivery, owing to their biomimetic lipid bilayer structure, tunable size (70–200 nm), and enhanced permeability and retention (EPR) effect, which promotes tumor-targeted drug accumulation [2]. However, a critical challenge remains: achieving quantitative control over drug encapsulation while preserving SUV structural integrity—a dual prerequisite for clinical translation [3,4].

Current strategies to modulate SUV permeability fall into two categories: chemical and physical methods. Chemical approaches, such as pH-gradient loading [5] and molecular inductors [6], bypass energy input but face inherent limitations. For example, Cavalcante et al. (2021) achieved 90% encapsulation of doxorubicin in pH-gradient SUVs, yet this method failed to load non-ionizable drugs like paclitaxel [7]. Similarly, Wei et al. (2020) demonstrated that hydrophobic inductors (e.g., polymyxin B) enabled 73.7% loading efficiency for curcumin, but the study did not evaluate potential cytotoxicity or biocompatibility in vivo, limiting its biomedical applicability [8]. These payload restrictions and safety risks hinder further biomedical applications. To address these challenges, physical methods—including thermal cycling [9,10], optoacoustic cavitation [11,12,13], and electroporation [14,15,16]—were developed transiently disrupt lipid bilayers through external energy inputs. For instance, Cardoso et al. demonstrated that thermal cycling of DPPC-based solid magneto liposomes creates transient pores at 42 °C, enabling heat-triggered doxorubicin release [17]. However, this approach lacks spatial precision and risks denaturing thermolabile payloads, as shown by Van den Broek et al. who observed that heat-stressed vesicles without heat shock protein protection suffered from protein misfolding and functional degradation [18]. Optoacoustic methods, while precise, depend on exogenous agents. Vaishnav and Mukherjee achieved localized membrane stress in SUVs using gold nanoparticles and near-infrared lasers, but residual nanoparticles persisted in 24% of vesicles, complicating biomedical workflows [19]. Furthermore, electroporation is widely adopted for rapid loading, but suffers from residual defects. Urakami et al. (2021) reported that SUVs underwent sustained deformation and membrane division due to lipid flip-flops induced by electroporation, leading to irreversible structural defects [20]. Collectively, these approaches aim to enhance drug encapsulation through diverse mechanisms, yet each faces trade-offs, such as irreversible vesicle damage, inconsistent membrane responses, or scalability challenges, that hinder further application.

Conventional microbubble-assisted ultrasound (MBUS) strategies enhance sonoporation through inertial cavitation but face limitations: (1) microbubble aggregation and uneven distribution compromise spatial precision and (2) inertial cavitation-induced risks, including cell damage and vesicle rupture **[21]**. Recent advances in acoustofluidics offer a transformative solution. Unlike conventional MHz-range sonoporation—which relies on microbubbles and risks inertial cavitation [22]—microelectromechanical system (MEMS)-based gigahertz (GHz) resonators generate cavitation-free acoustic streaming [23]. By leveraging hydrodynamic shear forces generated at high frequencies, GHz acoustic streaming can achieve programmable and nondestructive membrane permeabilization. Prior studies (e.g., ref. [23]) have visualized giant unilamellar vesicle (GUV) deformation under GHz acoustic streaming, demonstrating that shear stresses generated by micro-vortices create transient nanopores (~200 nm) in lipid membranes. Critically, these pores are reversible and do not compromise vesicle structural integrity. This technology induces reversible membrane remodeling (acoustomechanical permeabilization) via hydrodynamic shear forces, enabling programmable drug influx without structural damage. For example, Sedaghatkish et al. (2020) demonstrated that acoustic streaming, combined with thermosensitive liposomes, improved drug delivery efficiency by enhancing particle transport under high-frequency acoustic waves, highlighting its potential for non-invasive, targeted drug release [24]. Crucially, Lu et al. (2019) validated the reversibility of membrane permeabilization through real-time fluorescence imaging [25]. These studies highlight the ability of GHz acoustic streaming to harmonize high-efficiency loading with structural preservation.

In this study, we present a novel acoustofluidic strategy leveraging MEMS resonators (1.55 GHz) to achieve high-efficiency doxorubicin (DOX) encapsulation in lipid vesicles across size regimes. Crucially, DSPE-PEG synergizes with the encapsulation process to facilitate synthesis of dual-component (DOPC: DSPE-PEG) SUVs. As described in previous research, this amphiphilic lipid can enhance carrier stability and extend the circulation half-life [26,27]. Additionally, DSPE-PEG modulates membrane fluidity, suppressing membrane recovery during physical loading [28], while its pH-independent maleimide group minimizes nonspecific cellular interactions **[29]**. These properties position DSPE-PEG-modified SUVs as ideal candidates for advanced loading techniques. By systematically comparing two loading paradigms—(1) GUV pre-loading followed by downsizing vs. (2) direct SUV loading—we establish an optimized protocol that synergizes the high drug capacity of giant vesicles with the enhanced biodistribution of nanoscale carriers. The DSPE-PEG/acoustofluidic method achieved 60.04% encapsulation efficiency for DOX acitive loading while maintaining SUV integrity. Our findings provide a nondestructive drug encapsulation method, offering a potential strategy for carrier-based drug delivery in future.

## 2. Materials and Methods

### 2.1. Materials

1,2-Dioleoyl-sn-glycero-3-phosphocholine (DOPC) and Texas Red-modified 1,2-dihexadecanoyl-sn-glycero-3-phosphoethanolamine (Texas Red-DHPE) were purchased from Thermo Fisher Scientific Inc. (Waltham, MA USA). 1,2-Distearoyl-sn-glycero-3-phosphoethanolamine-N-[maleimide(polyethylene glycol)-2000] (ammonium salt) (DSPE-PEG) was obtained from Shanghai Macklin Biochemical Co. (Shanghai, China). Doxorubicin, chloroform, and an extrusion set with 100 nm pore-size polycarbonate filter membranes were purchased from Avanti Polar Lipids (Alabaster, AL, USA). All lipids were stored in chloroform at −20 °C and used without further purification.

### 2.2. Preparation of GUVs

Giant unilamellar vesicles (GUVs) were prepared using an electroformation method. The unlabeled lipid mixture consisted of DOPC and DSPE-PEG at a molar ratio of 95:5, while the fluorescence-labeled mixture included Texas Red-DHPE, with a molar ratio of 95:4.5:0.5. The mixed lipid solution was drop-cast onto the conductive side of an indium tin oxide (ITO) glass slide, and the solvent was evaporated using a nitrogen stream to form a uniform lipid film (see Figure S1 in Supplementary Information). The slide was then placed in a vacuum desiccator overnight. After drying, the lipid-coated slide was positioned on top of a clean ITO glass slide, with the conductive surfaces facing each other, and sealed with a rubber ring. The chamber between the slides was filled with 300 μL of deionized water. The slides were secured in a clamp, and GUVs were generated using a commercial vesicle preparation instrument (Nanion Technologies GmbH, Munich, Germany) under the following conditions: 5 Hz sinusoidal wave, peak voltage of 3 V, and a continuous operation time of 160 min. After formation, the GUVs were extracted from the chamber using a pipette, stored in plastic tubes, and kept at 4 °C.

### 2.3. MEMS Resonator Fabrication

The MEMS resonator was fabricated using a standard MEMS microfabrication process. Its multilayered structure comprises a Bragg reflector substrate, with a typical piezoelectric resonator layer deposited on top. This piezoelectric layer consists of two molybdenum (Mo) electrodes (top and bottom) and a central aluminum nitride (AlN) piezoelectric layer, which generates acoustic streaming in solution. The materials and structural dimensions of the MEMS resonator are similar to those reported in [23]. Testing with a vector network analyzer (FSVA3030 Signal and Spectrum Analyzer, Rohde & Schwarz GmbH & Co. KG, Munich, Germany) confirmed that the device’s resonant frequency is approximately 1.55 GHz. Consequently, the power amplifier was configured to operate at 1.55 GHz during experiments.

### 2.4. Acoustic Experimental Setup

To assemble the acoustic setup, a PDMS ring (1.5 cm inner diameter, 5 mm height) was placed atop the resonator. The PDMS ring used in the acoustic experimental setup was fabricated in the lab. Specifically, (1) PDMS (Dow Corning Sylgard 184) was prepared by mixing the base and curing agent at a 10:1 weight ratio. The mixture was thoroughly stirred with a glass rod for 10 min to ensure uniform mixing. It was then degassed under vacuum for approximately 30 min to remove trapped air bubbles. (2) The mixture was poured into a cylindrical container, and cured at 80 °C for 2 h to form a solid PDMS block. (3) After curing, the PDMS block was mechanically punched using circular cutters to define the ring geometry (2.5 cm outer diameter, 1.5 cm inner diameter). A 100 µL of GUV suspension and 100 µL of DOX aqueous solution (80 µg/mL) were added separately. Sinusoidal signals at 1.55 GHz were generated using a signal generator (SSG-6000RC Signal Generator, Mini-Circuits, Brooklyn, NY, USA), amplified by a power amplifier (ZHL-5W-422 Power Amplifier, Mini-Circuits, Brooklyn, NY, USA), and applied to the device. The electrical input was controlled by the software provided with the signal generator, which was used to turn the acoustic device on and off (see Figure S2 in Supplementary Information).

### 2.5. Preparation of DOX-Encapsulated SUVs

The GUV suspension was mixed with the DOX solution by gentle shaking at approximately 60 rpm for 2 min and then transferred to the PDMS chamber. The device’s working power was adjusted by tuning the gain settings of the signal generator, including acoustic streaming at varying intensities in the mixed solution. Under the influence of acoustic streaming, the permeability of the GUV membrane was enhanced, allowing DOX to enter into the vesicles.

Two strategies were employed to prepare DOX-loaded SUVs: (1) GUV-to-SUV Method: DOX-loaded GUVs were extruded through a 100 nm polycarbonate filter membrane 11 times using a liposome extruder (Avanti Polar Lipids), producing SUVs with a diameter of approximately 100 nm encapsulating DOX. The SUV suspension was then dialyzed against ultrapure water for 12 h using a magnetic stirrer to remove unencapsulated DOX. The purified DOX-loaded SUV suspension was collected and stored in plastic tubes. (2) Direct SUV Loading: Empty GUVs were first extruded through a 100 nm polycarbonate filter membrane to form SUVs. The resulting SUV suspension was then mixed with DOX in the PDMS chamber, allowing direct DOX loading into the SUVs.

### 2.6. Absorbance Measurements

To assess the impact of acoustic power on DOX encapsulation efficiency, GUV suspensions were mixed with DOX (80 μg/mL) and exposed to acoustic stimulation at varying input power levels for 10 min with acoustic stimulation applied for 10 min at a fixed frequency of 1.55 GHz: 0 mW (control), 100 mW, 150 mW, 200 mW, 230 mW, and 250 mW. Each condition was repeated three times. Following acoustic stimulation, samples underwent dialysis (12 h, ultrapure water of 18.2 MΩ·cm) to remove residual DOX. A single dialysis step was performed post-treatment and prior to absorbance measurements to ensure removal of free DOX. To confirm this process, we measured the DOX concentration in the external dialysate after the 12 h period. It revealed negligible residual DOX (Figure S6). The absorbances of DOX in these DOX-loaded vesicle samples were then measured at 490 nm (Elx808 Absorbance Microplate Reader, BioTek Instruments, Winooski, VT, USA).

### 2.7. TEM Measurements

TEM imaging was used to analyze the morphology of SUV carriers pre- and post-DOX loading. For negative staining, a 10 µL aliquot of the DOX-SUV sample was placed onto a copper grid for 1 min, and excess liquid was removed using filter paper. Next, 10 µL of uranyl acetate solution was added to the grid and allowed to settle for 1 min, followed by removal of excess staining solution. The sample was air-dried at room temperature before TEM observation. Imaging was performed using a HITACHI HT7800 transmission electron microscope operating at 80–120 kV. The obtained images were analyzed to assess the morphology and size distribution of DOX-SUVs. Vesicle size distribution and morphology analysis were performed using ImageJ software (version 1.54f, National Institutes of Health, Bethesda, MD, USA).

### 2.8. Statistical Analysis

Three independent experiments (*n* = 3) were conducted for the following analyses, including (1) DOX encapsulation efficiency in GUV systems across acoustic power levels (100 mW, 150 mW, 200 mW, 230 mW, 250 mW); (2) DOX encapsulation efficiency in SUVs under identical acoustic power conditions; and (3) comparative assessment of encapsulation efficiency between the GUV-first and SUV-direct loading strategies at matched power levels. Linear analysis was employed to analyze the relationship between acoustic input power and DOX encapsulation efficiency. A comparative analysis between the GUV-first and SUV-direct loading strategies at each acoustic power level was conducted using Student’s *t*-test. Statistical significance was defined as *p* < 0.05 (Supplementary Information, Section S4.3). All analyses were performed using Origin software (version 2021, OriginLab Corporation, Northampton, MA, USA).

## 3. Results and Discussion

### 3.1. Acoustic Streaming-Driven Drug Loading Platform

**Figure 1:**
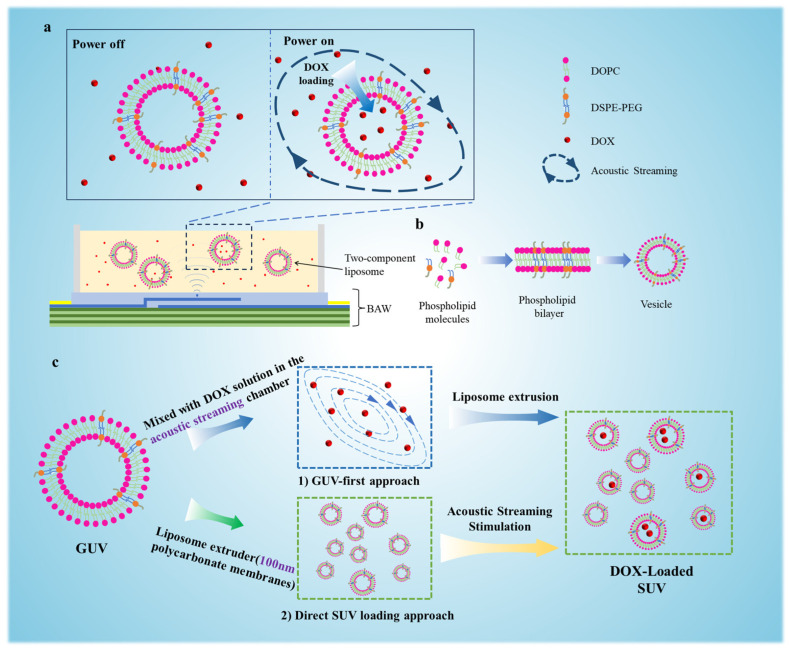
Schematic representation of drug loading into vesicles with assistance of acoustic streaming. (**a**) The drug loading platform based on acoustic streaming generated from a MEMS resonator operating at 1.55 GHz. (**b**) Assembly of two-component (DOPC:DSPE-PEG) GUVs. (**c**) A comparative analysis of two distinct loading strategies was conducted: (1) GUV-first approach: DOX encapsulation into GUVs followed by extrusion through 100 nm polycarbonate membranes to yield SUVs vs. (2) direct SUV loading: Acoustic delivery of DOX into pre-formed SUVs.

As schematically illustrated in Figure 1a, we developed a MEMS-based high-frequency acoustic resonator to generate controlled acoustic streaming within an aqueous medium. This platform enabled the successful encapsulation of DOX into vesicles composed of a binary lipid formulation (DOPC:DSPE-PEG, Figure 1b). Throughout the loading process, vesicle structural integrity was rigorously preserved. Following termination of acoustic stimulation, the vesicle stayed intact. This reversible permeabilization mechanism facilitated the formation of intact DOX-loaded liposomal carriers with high reproducibility.

As shown in Figure 1c, the experimental workflow comprised two sequential phases. Initially, drug loading was investigated in GUVs (diameter > 10 μm) to enable direct visualization and quantitative analysis of DOX encapsulation efficiency under varying acoustic powers (0–250 mW). Subsequently, the protocol was adapted for SUVs (diameter~100 nm), and a comparative analysis of two distinct loading strategies was conducted: (1) GUV-first approach: DOX encapsulation into GUVs followed by extrusion through 100 nm polycarbonate membranes to yield SUVs vs. (2) direct SUV loading: Acoustic delivery of DOX into pre-formed SUVs.

### 3.2. Synthesis and Structural Characterization of GUVs

The synthesis of GUVs was achieved through a refined electroformation protocol optimized for binary lipid systems. As illustrated in Figure S1, a homogeneous lipid mixture comprising DOPC and DSPE-PEG at a 95:5 molar ratio was dissolved in chloroform and meticulously deposited onto indium tin oxide (ITO)-coated glass substrates. The solvent was evaporated under a nitrogen stream to form a uniform lipid film, followed by overnight vacuum desiccation to eliminate residual solvent. Prior to hydration, the lipid film’s homogeneity was verified via fluorescence microscopy after incorporating 0.5 mol% Texas Red-DHPE, a lipophilic fluorescent probe. Electroformation was conducted in a custom hydration chamber assembled from two ITO slides separated by a silicone spacer. A 300 μL aliquot of deionized water (18.2 MΩ·cm) was injected into the chamber, and an alternating current (AC) electric field (10 Hz, 3 V peak-to-peak) was applied for 120 min at 30 °C. This AC field facilitated lipid bilayer bending and self-assembly into GUVs through a combination of electrophoretic forces and mechanical stress [30].

**Figure 2:**
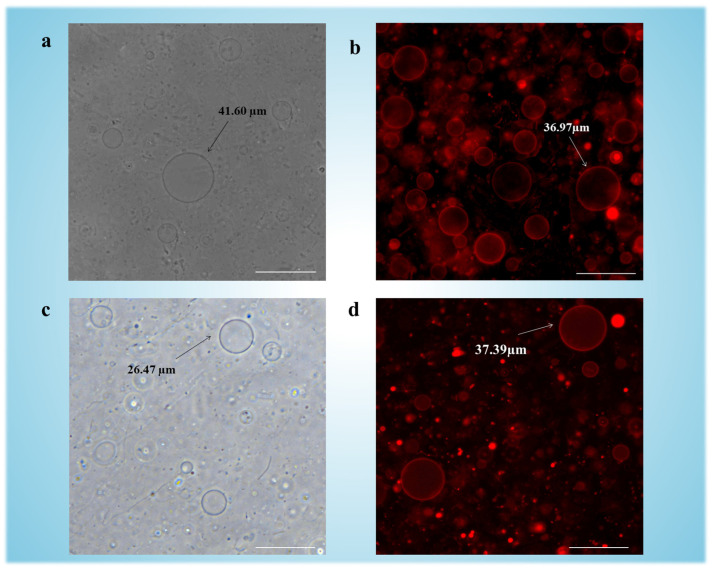
Images of electroformed GUVs: (**a**) Phase contrast imaging of GUVs made of DOPC:DSPE-PEG (ratio: 95:5). (**b**) Fluorescence imaging of GUVs made of DOPC:DSPE-PEG: Texas-Red-DHPE (ratio: 95:4.5:0.5). (**c**) Phase contrast imaging of GUVs made of pure DOPC. (**d**) Fluorescence imaging of GUVs made of DOPC: Texas-Red-DHPE (ratio: 95:4.5:0.5). Scale bars indicate 50 µm. In fluorescence images, red fluorescence indicates the presence of Texas-Red-DHPE incorporated into the GUV membranes.

In Figure 2a, phase-contrast microscopy confirmed the formation of GUVs with diameters exceeding 40 μm, while fluorescence imaging (Figure 2b) facilitated by Texas Red-DHPE (0.5 mol%) within the bilayers confirmed intact vesicle morphology. Notably, plasma treatment of ITO slides prior to lipid deposition enhanced surface hydrophilicity, promoting uniform hydration and yielding an increased number of GUVs (see Figure S3 in Supplementary Information) compared to untreated controls [31].

To evaluate the role of DSPE-PEG in GUV formation, parallel experiments were conducted with pure DOPC vesicles (Figure 2c). The low main phase transition temperature (T_m_ = −17 °C [32]) of DOPC rendered its bilayers prone to phase separation under mechanical stress, resulting in heterogeneous vesicle populations (1–40 μm diameter) with low colloidal stability. In contrast, DSPE-PEG-modified GUVs exhibited monodisperse size distributions (20–50 μm), with most of vesicles retaining structural integrity for >48 h post-synthesis (Figure S4 in Supplementary Information). This enhancement is attributed to PEG2000’s steric stabilization, which suppresses bilayer buckling and coalescence during electroformation [33,34]. The fluorescence imaging is shown in Figure 2d.

The binary DOPC:DSPE-PEG formulation demonstrated superior performance over pure DOPC across three critical parameters: yield (717 vesicles/mm^2^ vs. 326 vesicles/mm^2^), stability, and size uniformity (polydispersity index [PDI] of 0.318 vs. 0.523, see calculation parts corresponding to Figure S5 in Supplementary Information). These enhancements reflect DSPE-PEG’s role in stabilizing lipid bilayers through fluidity modulation, which suppressed vesicle coalescence during electroformation while maintaining structural integrity post-synthesis. The improved monodispersity and colloidal stability are particularly vital for standardizing vesicle populations in drug loading applications, where consistent size-dependent encapsulation efficiency and carrier longevity are prerequisites for reproducible therapeutic outcomes.

### 3.3. Power-Dependent DOX Loading in GUVs

In this study, we engineered GUV carriers (>10 μm diameter) composed of a binary lipid formulation (DOPC:DSPE-PEG) to investigate the relationship between the input power of acoustic streaming and DOX encapsulation efficiency. While GUVs of this size are not suitable for drug delivery [35], their large aqueous lumen and visual accessibility make them ideal model systems for quantifying drug loading mechanisms [36,37]. To assess DOX encapsulation, pre-formed GUVs were mixed with an 80 μg/mL DOX solution in a PDMS microchamber integrated with a 1.55 GHz MEMS resonator.

**Figure 3:**
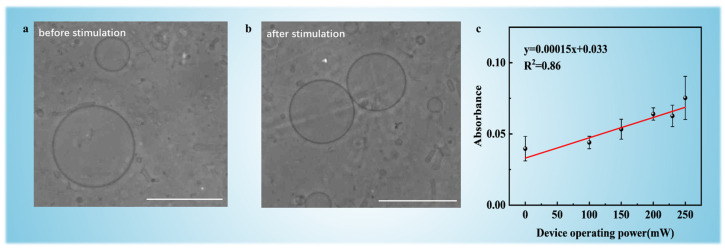
Phase-contrast microscopy images of GUVs (**a**) before and (**b**) after acoustic streaming stimulation (250 mW, 10 min). Scale bars indicate 50 µm. (**c**) Absorbance of DOX-loaded GUVs as a function of acoustic stimulation at different input power levels (0, 100, 150, 200, 230, and 250 mW).

Acoustic streaming was generated at incremental power levels (100, 150, 200, 230, and 250 mW) for 10-min durations. Hydrodynamic shear stresses induced by GHz-frequency acoustic streaming created transient membrane deformations, forming nanopores that permitted DOX influx while maintaining overall vesicle integrity [23]. In Figure 3a,b, phase-contrast microscopy confirmed structural preservation of GUVs after the stimulation of acoustic streaming at 250 mW for 10 min, with most GUVs retaining spherical morphology post-stimulation.

Figure 3c reveals a linear trend of DOX loading efficiency with increasing acoustic power from 0 to 250 mW. The absorbance of DOX-loaded GUVs at λ = 490 nm was calibrated against the absorbance of 40 μg/mL DOX solution (reference value) to determine the drug loading efficiency under different power levels (Figure S7 see details in Supplementary Information). Encapsulation efficiency increased progressively from 33.17 ± 2.1% at 100 mW to 58.03 ± 1.8% at 250 mW, demonstrating power-dependent control over membrane permeability. This trend is attributed to intensified acoustic shear forces at higher power, which is likely to increase pore density and duration without exceeding the critical stress threshold for bilayer rupture.

Mechanistically, DSPE-PEG played a dual role in this process. The PEG2000 side chains contributed to fluidization by reducing bilayer rigidity, thereby lowering the energy barrier for pore formation under shear stress [38]. Additionally, DSPE-PEG facilitated self-repair through steric repulsion between PEG moieties, which accelerated pore closure kinetics [26]. Notably, extrapolation beyond 250 mW revealed a trade-off: while higher power could theoretically enhance loading, it risked exceeding the lipid bilayer’s critical tension (≈4 mN/m for DOPC [39]), leading to irreversible rupture (300 mW; see Figure S8 in in Supplementary Information). This establishes 250 mW as the optimal operational limit for balancing efficiency (58.03% ± 1.8%) with structural preservation (most GUVs have integrity).

The linear power–efficiency relationship provides a predictive framework for customizing drug payloads in liposomal systems. By modulating acoustic parameters (power, duration) alongside lipid composition (DOPC, DSPE-PEG molar ratio), this platform enables precise tuning of encapsulation efficiency, which is a critical advantage for dose-dependent therapeutics. These findings validate GHz acoustic streaming as a useful tool for nondestructive vesicle engineering, with implications for optimizing carrier designs in targeted drug delivery and personalized nanomedicine. Future research will investigate alternative lipid compositions, such as high-T_m_ lipids (e.g., DPPC, DSPC) or cholesterol-stabilized membranes, to enhance bilayer shear stress resilience under acoustic streaming. In parallel, resonator design optimization, including tuning acoustic frequency or refining device geometry to homogenize the distribution of the acoustic streaming field, could mitigate membrane rupture. These combined strategies may extend the operational power range while enabling higher-throughput processing, thereby advancing platform versatility for diverse therapeutic cargo and lipid systems.

### 3.4. SUV Loading Efficiency Comparison

GUVs exceeding 10 μm in diameter exhibit rapid clearance by macrophages via the mononuclear phagocyte system [35]. In contrast, SUVs with nanoscale sizes (around 100 nm) evade phagocytic recognition, prolonging circulation half-life through enhanced permeation of hepatic capillary pores (diameter ~150 nm) [40]. Furthermore, SUVs exploit the tumor microenvironment’s pathophysiological features—notably the hyperpermeable vasculature and impaired lymphatic drainage characteristic of the enhanced permeability and retention (EPR) effect [41]. This passive targeting mechanism makes SUVs < 100 nm as ideal carriers for chemotherapeutic agents like DOX.

**Figure 4:**
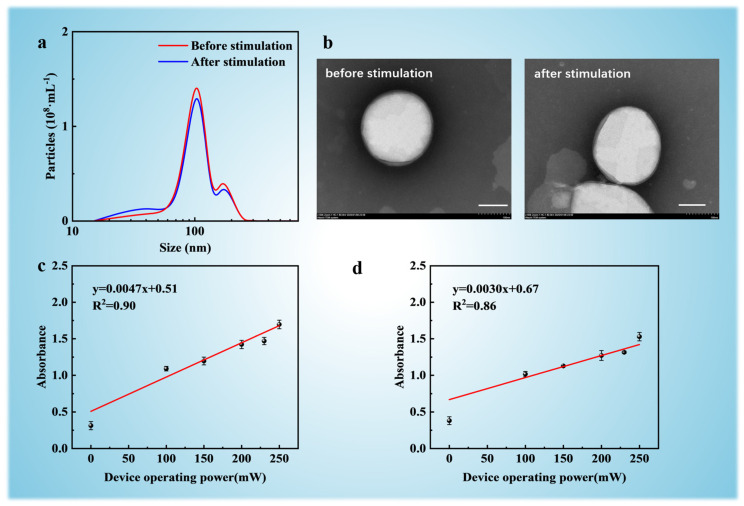
The experimental results of DOX loading into SUVs. (**a**) Particle size distribution measured by DLS before and after the stimulation of acoustic streaming (250 mW, 10 min). (**b**) TEM images of SUVs before and after acoustic streaming stimulation (250 mW, 10 min). Scale bars indicate 100 nm. Absorbance of DOX-loaded SUVs under acoustic streaming at different input power levels (0, 100, 150, 200, 230, and 250 mW) based on two methods. (**c**) DOX is first delivered to GUVs, which are then downsized into SUVs; (**d**) SUVs are prepared first, followed by direct DOX loading.

In this study, two distinct strategies were evaluated for preparing DOX-loaded SUVs: (1) GUV-first strategy: Pre-formed DOX-loaded GUVs were extruded through 100 nm polycarbonate membranes using a thermostabilized liposome extruder. This downsizing process yielded SUVs (100 ± 15 nm, Figure 4a) with encapsulated DOX. (2) Direct SUV loading: Empty SUVs were first prepared by extruding unloaded GUVs, followed by acoustic streaming-mediated DOX loading into pre-formed SUVs. Prior to drug loading directly to SUVs, vesicles morphology and size distribution were validated via transmission electron microscopy (TEM) and Nanoparticle Tracking Analysis (NTA). As shown in Figure 4b, TEM micrographs confirmed monodisperse spherical SUVs with a mean diameter of 100 nm, ensuring population homogeneity. Post-loading TEM analysis confirmed intact SUV morphology across both strategies, with no evidence of membrane rupture or aggregation.

For both strategies, DOX encapsulation was performed using a 1.55 GHz MEMS resonator at incremental power levels (100–250 mW). Absorbance measurements (λ = 490 nm) quantified DOX concentrations post-dialysis (see details in Supplementary Information). For strategy 1 (GUV-first, Figure 3c), DOX loading efficiency increased in a linear trend with acoustic power (R^2^ = 0.86), reaching 60.04% ± 1.55% at 250 mW. The larger aqueous volume of GUVs (>10 μm) provided a higher drug-accessible reservoir prior to extrusion, enabling superior payload retention during downsizing. Meanwhile, for strategy 2 (Direct SUV loading, Figure 4d), despite similar power-dependent trends, maximum encapsulation efficiency plateaued at 54.11% ± 0.72% (250 mW) due to the constrained internal volume of pre-formed SUVs (~100 nm), which limited DOX influx capacity.

The superior performance of the GUV-first strategy is likely to arise from two synergistic factors. First, GUVs offer a larger internal volume (taking a GUV with a diameter of 35 μm as an example, the volume is approximately 7.19 × 10^13^ nm^3^ vs. 5.24 × 10^5^ nm^3^ for SUVs), enabling greater drug accumulation before downsizing. Second, the PEG2000 corona minimizes payload leakage during extrusion, significantly reducing DOX loss compared to PEG-free controls [42]. These findings provide an optimized strategy for nanocarrier engineering—leveraging micron-scale vesicles for high-capacity loading followed by downsizing to obtain nanoscale biodistribution. The scalability possibility of this methodology, enabled by MEMS-compatible acoustic streaming systems, will provide a potential method for efficient production of drug carriers.

## 4. Conclusions

This study established MEMS-driven GHz acoustofluidics as a platform for nondestructive, high-efficiency drug encapsulation into lipid vesicles, addressing critical limitations of conventional carrier engineering methods. By leveraging cavitation-free acoustic streaming (1.55 GHz), we achieved controlled delivery of DOX into both GUVs and SUVs while maintaining structural integrity. The integration of DSPE-PEG proved pivotal: its steric stabilization and fluidizing effects enabled transient nanopore formation under hydrodynamic shear stress from GHz acoustic streaming. Comparative analysis of two loading strategies revealed the superiority of the GUV-first approach, achieving 60.04% encapsulation efficiency at 250 mW, outperforming direct SUV loading (54.11%). This disparity arises from GUVs’ larger aqueous volume, which enhances drug-accessible capacity prior to extrusion. By combining carrier fabrication (electroformation/extrusion) from drug loading (acoustic streaming), this research provides a potential strategy for nanocarrier production in drug delivery. Future research will prioritize biological validation, including in vitro and in vivo assessments of therapeutic efficacy and biodistribution, to advance this platform toward clinical applications.

## Data Availability

The original contributions presented in this study are included in the article. Further inquiries can be directed to the corresponding authors.

## Supplementary Materials

The following supporting information can be downloaded at: https://www.mdpi.com/article/10.3390/mi16050562/s1, Figure S1: The electroformation setup of GUVs and the corresponding geometric parameters of the device; Figure S2: Schematic of the experimental setup; Figure S3: (a) Vesicles without plasma treatment. (b) Vesicles with plasma treatment; Figure S4: GUVs observed at 0, 24, 48, 96, and 168 h after electroformation; Figure S5: Images of GUVs with two different compositions [43]; Figure S6: Absorbance (a.u.) of dialysate samples after different durations of dialysis (3–12 h) and ultrapure water (Control); Figure S7: DOX levels in GUV mixtures after acoustic treatment (150–300 mW), relative to 40 µg/mL reference; Figure S8: Higher acoustic power led to lower absorbance, suggesting reduced DOX encapsulation; Table S1: The specific absorbance values of DOX-GUVs after acoustic streaming treatment, along with the reference values.
