# Supplementary material for: High-Efficiency Drug Loading in Lipid Vesicles by MEMS-Driven Gigahertz Acoustic Streaming
Source: Micromachines (Basel). 2025 May 7;16(5):562. doi: 10.3390/mi16050562 (PMC12114090; doi:10.3390/mi16050562)
Supplement: Supplementary file 1 [file micromachines-16-00562-s001.zip › micromachines-3590086-supplementary.docx]

Supporting Information

High-Efficiency Drug Loading in Lipid Vesicles by MEMS-Driven Gigahertz Acoustic Streaming

Bingxuan Li ^1,2^, Haopu Wang ^1,2^, Zhen Wang ^1,2,3^, Huikai Xie ^1,2,3,^* and Yao Lu ^1,2,3,^*

^1^ School of Integrated Circuits and Electronics, Beijing Institute of Technology, Beijing 100081, China

^2^ Engineering Research Center of Integrated Acoustic-Opto-Electronic Microsystems (Ministry of Education of China), Beijing 100081, China

^3^ Chongqing Institute of Microelectronics and Microsystems, Beijing Institute of Technology,
Chongqing 400030, China

***** Correspondence: hk.xie@bit.edu.cn (H.X.); y.lu@bit.edu.cn (Y.L.)

1. Experimental Setup


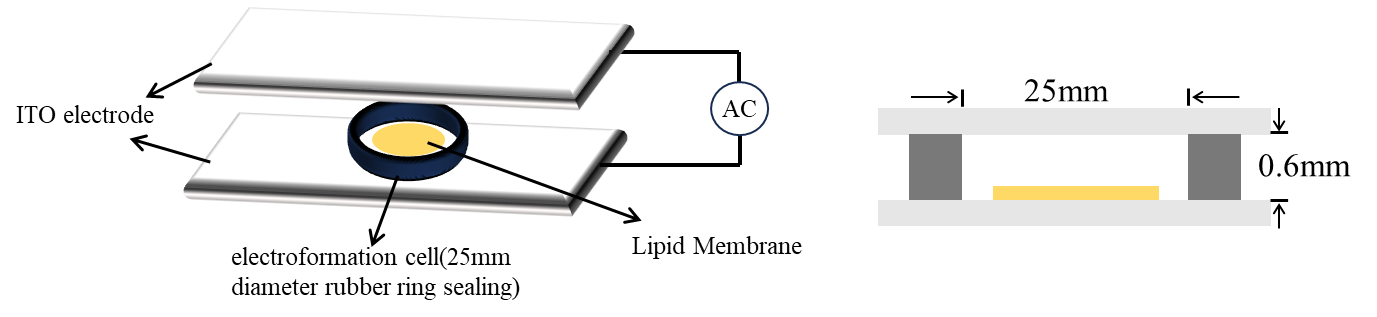


**Figure S1.** The electroformation setup of GUVs and the corresponding geometric parameters of the device.


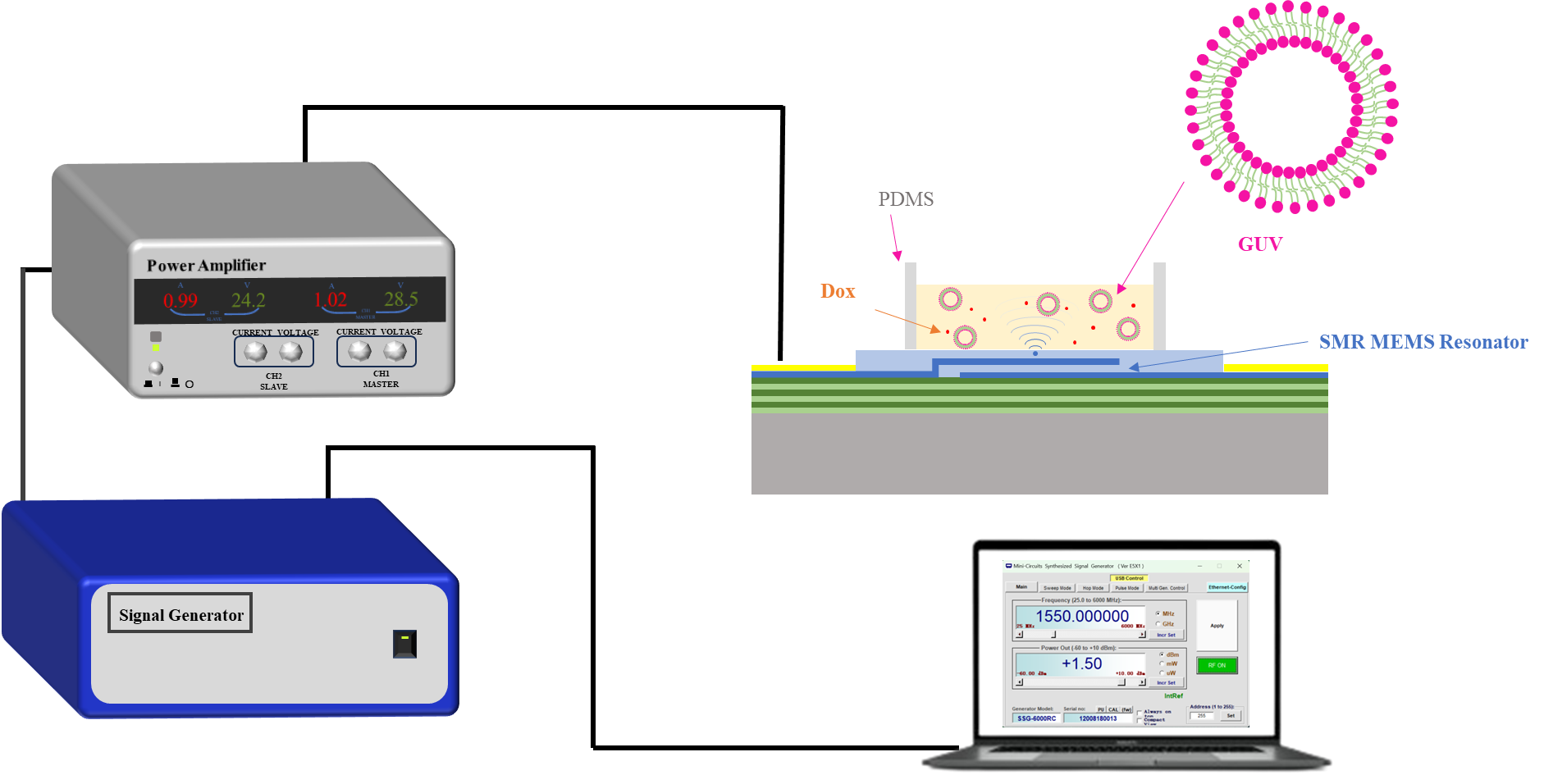


**Figure S2.** Schematic of the experimental setup: The electrical input was controlled via computer software to turn the acoustic device on and off, thereby regulating acoustic streaming and acoustic stimulation duration.

2. Synthesis and Structural Characterization of GUVs


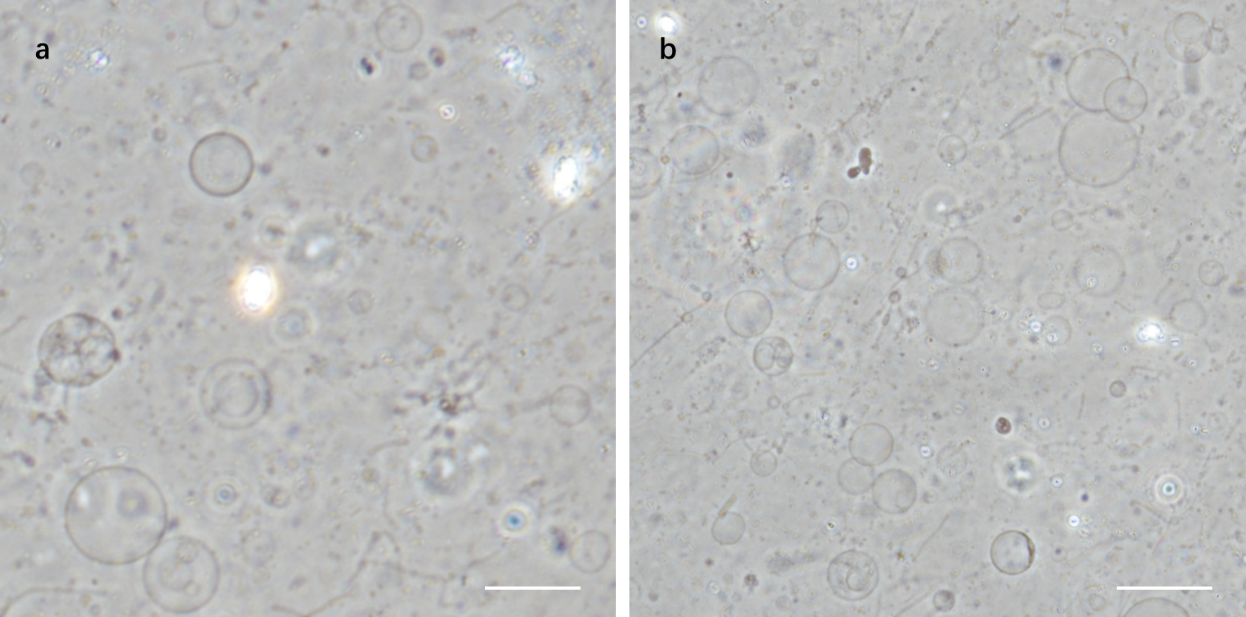


**Figure S3.** (a) DOPC:DSPE-PEG vesicles prepared without Plasma treatment. (b) DOPC:DSPE-PEG vesicles prepared with Plasma treatment. Plasma treatment of ITO slides prior to lipid deposition enhanced surface hydrophilicity, promoting uniform hydration and yielding a more than 50% increase in GUV density compared to untreated controls. Scale bars indicate 50 µm. All the vesicle size and number distribution and morphology analyses were performed using *ImageJ* software.


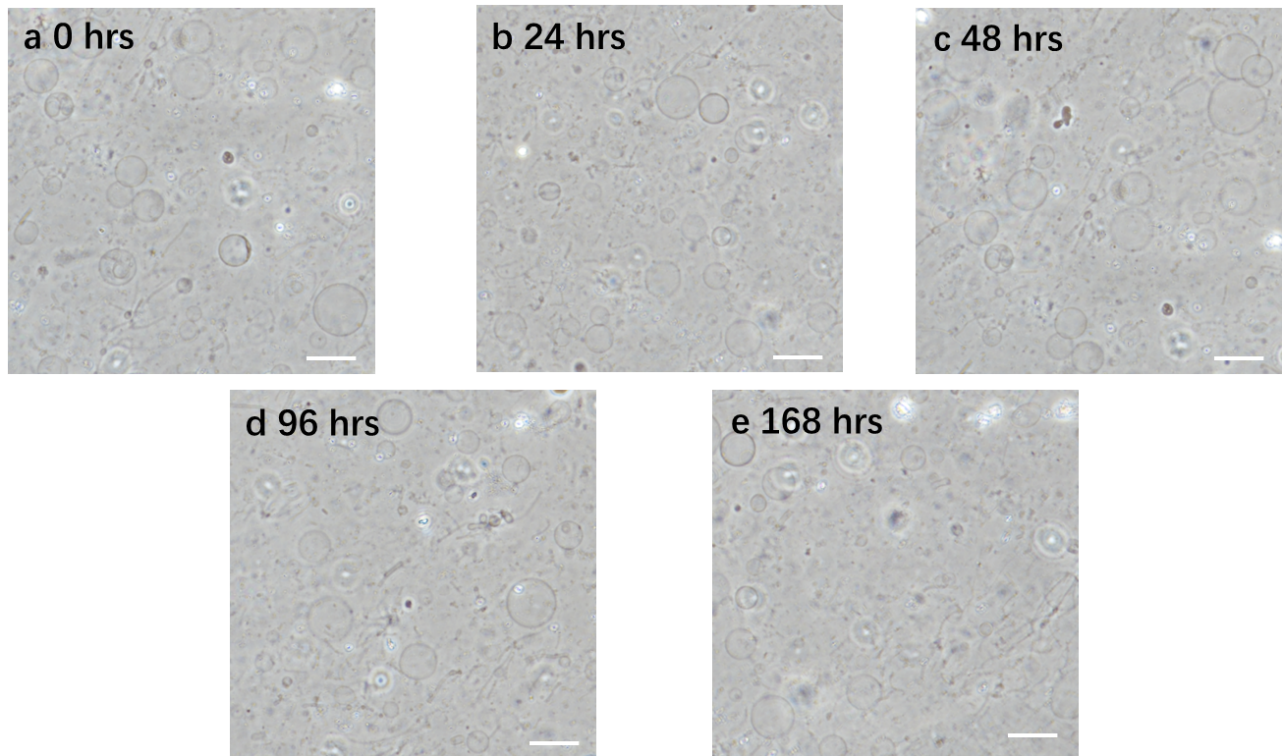


**Figure S4.** The observed GUVs after a period of time (0, 24, 48, 96, 168 hrs) following electroformation. Scale bars indicate 50 µm.

The numbers of GUVs in Figure S4 are listed in the table below:

| **Time (hrs.)** | 0 | 24 | 48 | 96 | 168 |
| --- | --- | --- | --- | --- | --- |
| **Num. of GUVs in Figure S4)** | 25 | 21 | 20 | 15 | 10 |

The GUVs in Figure S4 were obtained using 20x microscopy. To determine the total GUV count, we aggregated data from 10 distinct microscopic regions within the field of view. After applying 250 mW acoustic stimulation for 10 minutes, the same 10 regions were re-imaged under identical microscope settings to quantify post-stimulation GUV numbers. This protocol was repeated five times. The GUV numbers before and after treatment were similar, indicating most GUVs remained intact after acoustic treatment.


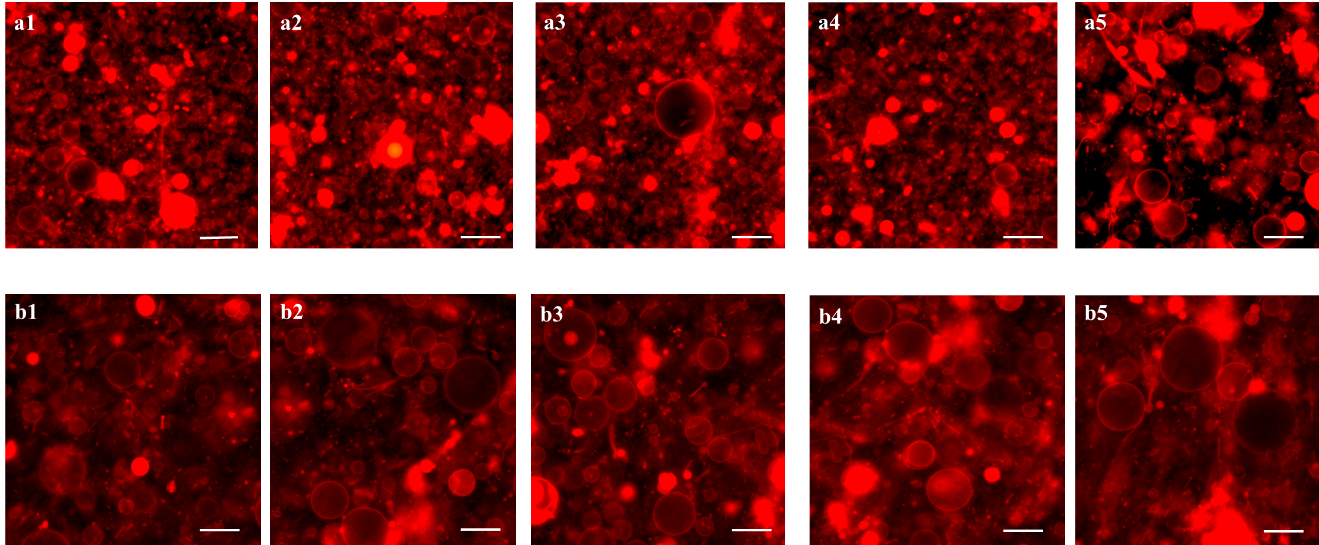


**Figure S5.** Images of GUVs with two different compositions. **a1-a5**: Pure DOPC; **b1–b5**: DOPC:DSPE-PEG. Scale bars indicate 50 µm.

For the two types of GUVs (DOPC:DSPE-PEG and pure DOPC), the average diameter and the number were calculated based on 10 fluorescence microscopy images. The PDI was determined using the following formula [1]:

|  |  |
| --- | --- |

The average diameter was obtained by measuring the diameters of all visible GUVs in Figure S5, which corresponds to the 0-hour time point immediately after vesicle synthesis. The standard deviation was calculated based on the distribution of these individual diameter measurements using the standard statistical formula. All measurements were conducted using ImageJ software, and the results represent the initial size distribution of GUVs prior to any acoustic treatment or extrusion.

1. DPOC: DSPE-PEG: Each image had an area of 82,229 μm², with an average count of 58 GUVs, an average diameter of 36.84 μm, a standard deviation of 20.77 μm, and a PDI of 0.318.
2. Pure DOPC: Each image had an area of 82,229 μm², with an average count of 25 GUVs, an average diameter of 25.48 μm, a standard deviation of 26.75 μm, and a PDI of 0.523.

3. Power-Dependent DOX Loading in GUVs

Calculation of DOX-GUV Encapsulation Efficiency

An aqueous DOX solution at a concentration of 80 µg/mL was mixed with an equal volume of GUVs suspension to obtain a final mixture with 40 µg/mL DOX. The initial absorbance of the DOX and GUVs mixture was measured at 490 nm and used as a reference (termed b), representing the total DOX amount in the system before acoustic treatment. Subsequently, the mixture was subjected to acoustic stimulation for 10 minutes at different input powers (100 mW, 150 mW, 200 mW, 230 mW, and 250 mW). After acoustic treatment, the mixtures in each condition underwent a 12-hour dialysis against ultrapure water to remove unencapsulated DOX molecules. To confirm this process, we measured the DOX concentration in the external dialysate after the 12-hour period. Results revealed negligible residual DOX (< 0.5% of initial concentration, Figure S6), demonstrating that a single dialysis step sufficiently removed unbound drug. The dialyzed GUVs were then collected, and their absorbance at 490 nm was measured (termed a), which was regarded as the amount of DOX successfully encapsulated inside the GUVs.

The encapsulation efficiency (EE%) was calculated according to the following equation:

|  |  |
| --- | --- |

where *a* is the absorbance of the dialyzed GUV suspension and *b* is the reference absorbance of the initial mixture. The results showed a gradual increase in DOX encapsulation efficiency with increasing acoustic power. Specifically, the EE% was 33.08% at 100 mW, 40.08% at 150 mW, 48.12% at 200 mW, 47.14% at 230 mW, and reached the highest value of 56.58% at 250 mW. To confirm the repeatability of the encapsulation process, the experiment at 250 mW was repeated five times independently, yielding a delivery efficiency consistently between 56.23% and 58.94%, with a standard deviation (SD) of ±1.8%.

**Table S1.** The specific absorbance values of DOX-GUVs after acoustic streaming treatment, along with the reference values.

| **Power** | **absorbance1** | **absorbance2** | **absorbance3** |
| --- | --- | --- | --- |
| 100 | 0.042 | 0.049 | 0.041 |
| 150 | 0.06 | 0.054 | 0.046 |
| 200 | 0.061 | 0.069 | 0.062 |
| 230 | 0.068 | 0.066 | 0.054 |
| 250 | 0.058 | 0.086 | 0.082 |
| reference | 0.131 | 0.133 | 0.134 |


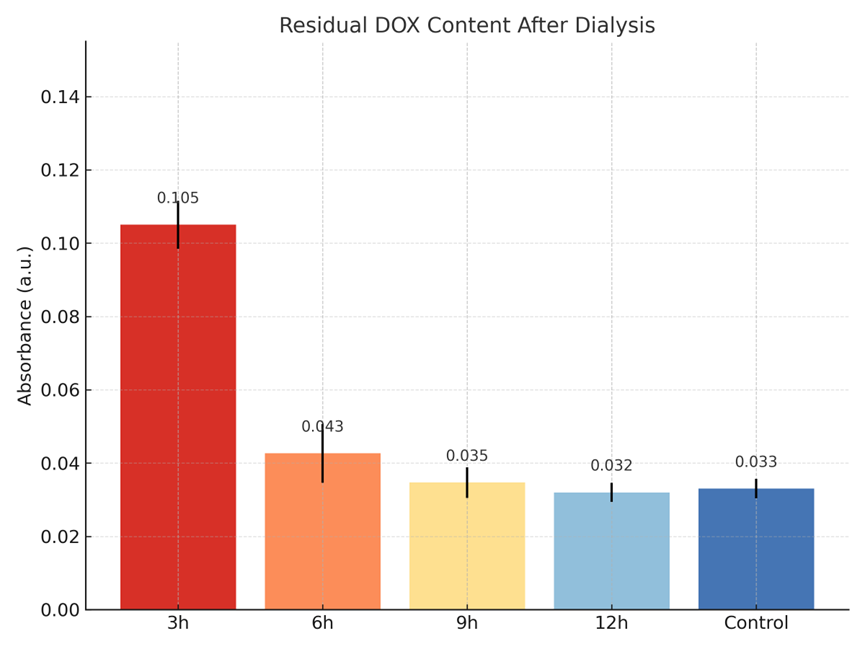


**Figure S6.** Absorbances (a.u.) of dialysate samples after different durations of dialysis (3–12 h) and ultrapure water (Control). The data indicate progressive removal of unencapsulated DOX over time. After 12 hours, the absorbance approaches that of ultrapure water.


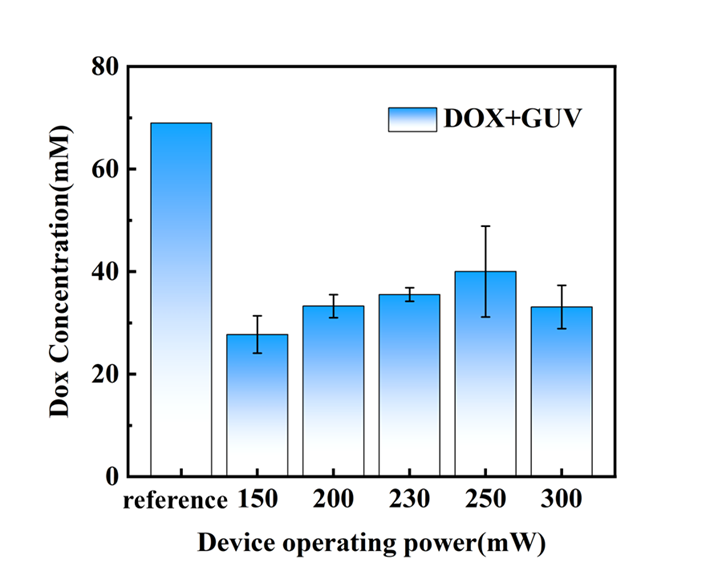


**Figure S7.** Compared to the 40 μg/mL DOX (reference), the DOX concentrations of DOX-GUV mixtures after treatment with different acoustic powers (150, 200, 230, 250 mW, and 300 mW).


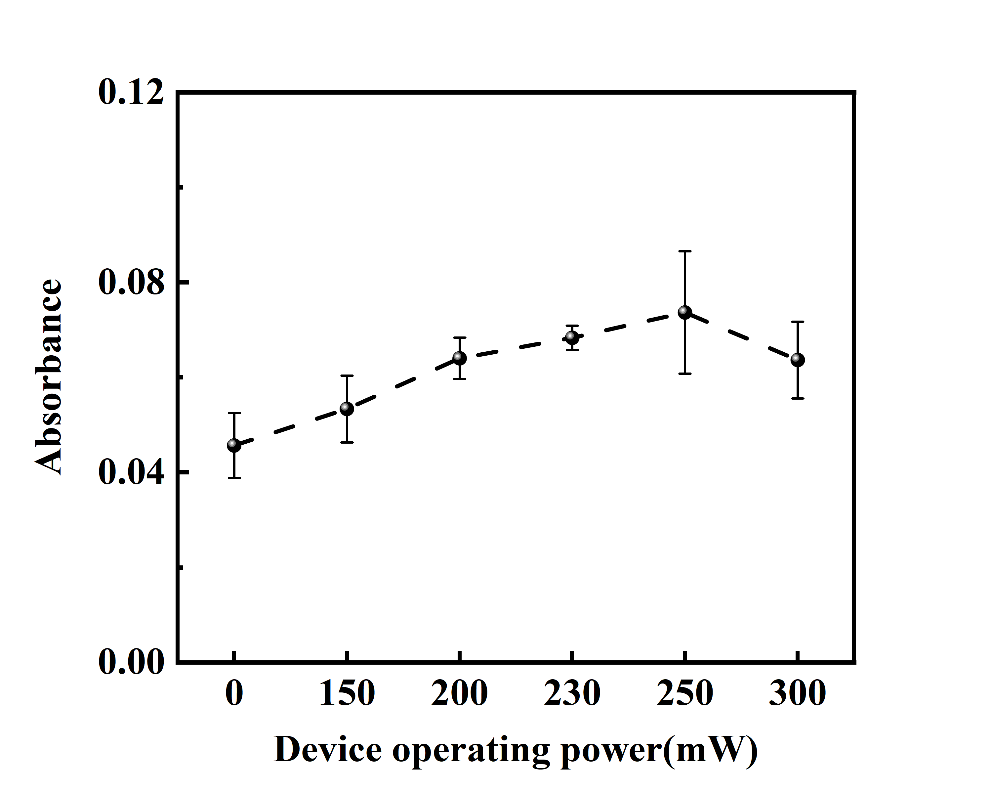


**Figure S8.** As the acoustic streaming power increased to 300 mW, the average absorbance of the DOX-loaded GUVs decreased, indicating a reduction in the amount of DOX encapsulated within the GUVs.

4. SUV Loading Efficiency Comparison

4.1. The process of obtaining DOX-SUV.

Two different strategies were employed to obtain DOX-loaded small unilamellar vesicles (SUVs) using GHz acoustic stimulation:

(1) GUV-first Strategy:

GUVs were first prepared and mixed with an equal volume of DOX solution (final DOX concentration: 40 µg/mL). The mixture was subjected to acoustic stimulation at specified input power levels for 10 minutes. After acoustic treatment, the resulting DOX-loaded GUV suspension was extruded through a 100 nm polycarbonate membrane filter 11 times to downsize the vesicles into SUVs (~100 nm diameter). The SUV suspension was then placed into a dialysis bag and dialyzed against ultrapure water for 12 hours under gentle stirring to remove free DOX molecules.

(2) SUV-direct Strategy:

GUVs were first extruded through a 100 nm polycarbonate membrane filter 11 times to generate empty SUVs (~100 nm diameter). These pre-formed SUVs were then mixed with an equal volume of DOX solution (final DOX concentration: 40 µg/mL) and immediately subjected to acoustic stimulation at specified input power levels for 10 minutes. The acoustic stimulation transiently enhanced the membrane permeability, allowing DOX to be loaded directly into the SUVs. Following treatment, the suspension was dialyzed against ultrapure water for 12 hours to remove free DOX.

In both strategies, the final DOX-loaded SUV suspensions were collected for subsequent absorbance measurements and encapsulation efficiency analysis.

4.2. Calculation of DOX-SUV Encapsulation Efficiency

Giant unilamellar vesicles (GUVs) were first prepared and mixed with an equal volume of DOX solution (final DOX concentration: 40 µg/mL). The mixture was then extruded through a 100 nm polycarbonate membrane filter 11 times to obtain a homogeneous suspension of SUVs and free DOX molecules. The absorbance of this SUV-DOX suspension was measured at 490 nm and taken as the reference value (b), representing the total DOX content in the system prior to acoustic stimulation. For the SUV-direct strategy, it would have been ideal to measure the SUV and DOX mixtures as a reference value. This simplification (using the same reference absorbance), used by considering the original DOX concentration in either DOX and SUV mixtures or DOX and GUV mixtures, would be similar due to the same mixed volume. The DOX-loaded SUV suspension was then subjected to acoustic stimulation at specified input power levels for 10 minutes. After treatment, the samples were dialyzed against ultrapure water for 12 hours to remove free, unencapsulated DOX molecules. The absorbance of the dialyzed SUV suspension was subsequently measured (a), reflecting the amount of DOX successfully encapsulated within the SUVs.

The encapsulation efficiency (EE%) was calculated using the following formula:

|  |  |
| --- | --- |

where *a* is the absorbance of the dialyzed SUV suspension after acoustic stimulation and dialysis, and *b* is the reference absorbance of the SUV-DOX mixture after extrusion but before acoustic stimulation.

For the GUV-first strategy, the DOX encapsulation efficiencies at different acoustic power levels were as follows: 38.64% at 100 mW, 42.35% at 150 mW, 50.35% at 200 mW, 52.02% at 230 mW, and 60.04% at 250 mW. The experiment was repeated five times at 250 mW, yielding encapsulation efficiencies between 59.15% and 61.59%, with a standard deviation (SD) of ±1.55.

For the SUV-direct strategy, the encapsulation efficiencies were 35.98% at 100 mW, 39.94% at 150 mW, 45.00% at 200 mW, 46.56% at 230 mW, and 54.11% at 250 mW. Repeated tests at 250 mW showed encapsulation efficiencies between 54.02% and 54.83%, with a standard deviation of ±0.72. All water-based dialysis and release experiments were conducted at room temperature (~25 °C).

4.3. Statistical comparison of GUV-first and SUV-direct strategies

To assess the statistical significance of the difference in drug encapsulation efficiency between the GUV-first and SUV-direct strategies at 250 mW input power, we performed an unpaired, two-tailed Student’s *t*-test.

The encapsulation efficiency values used for the comparison were obtained from five independent experiments for each group:

GUV-first strategy: [60.04%, 60.52%, 61.59%, 59.15%, 59.65%]

SUV-direct strategy: [54.11%, 54.02%, 54.83%, 54.55%, 54.23%]

Statistical analysis was performed using OriginPro 2021 (OriginLab Corporation, USA).

The resulting *p*-value was calculated to be *p* = 0.00023, indicating a statistically significant difference between the two strategies at the *p* < 0.05 level.

Reference

1. Flory, Paul J. Principles of polymer chemistry. Cornell university press, 1953.
